# Factors Associated With Positive Self-Rated Health: Comparing Older Adults in Brazil and in Portugal

**DOI:** 10.3389/fpubh.2021.650294

**Published:** 2021-03-29

**Authors:** Meire Cachioni, Gabriela Cabett Cipolli, Flávia Silva Arbex Borim, Samila Sathler Tavares Batistoni, Mônica Sanches Yassuda, Anita Liberalesso Neri, Constança Paúl

**Affiliations:** ^1^University of São Paulo, São Paulo, São Paulo, Brazil; ^2^Institute of Biomedical Sciences Abel Salazar (ICBAS), Center for Research in Health Technologies and Services (CINTESIS), University of Porto, Porto, Portugal; ^3^Graduate Studies in Gerontology, State University of Campinas, Campinas, São Paulo, Brazil

**Keywords:** self-rated health, neuroticism, old adults, Brazil, Portugal

## Abstract

**Introduction:** Self-rated health is a multidimensional health indicator and a predictor of adverse events in old age. Answers to this assessment are influenced by social, cultural and personality factors.

**Aim:** Exploring common and distinctive characteristics of Brazilian and Portuguese older adults aged 70 and over regarding positive self-rated health according to sociodemographic variables, to functional capacity, to independent performance of basic activities of daily living and to neuroticism, as well as analyzing associations between positive self-rated health and these variables.

**Methods:** The present paper is a comparative and cross-sectional study based on secondary data contained in the databases of the FIBRA (Frailty in Brazilian Older Adults) follow-up study, with 418 Brazilian older adults, and of the DIA (From Disability to Activity: The Challenge of Aging) study, with 380 Portuguese older adults. Both samples had higher percentages of women: 68.4% for Portugal and 69.9% for Brazil. The Brazilian sample had a higher average age (80.31 ± 4.67) than the Portuguese sample (76.80 ± 5.28).

**Results:** The Portuguese older adults had better overall cognition scores, higher handgrip strength and higher neuroticism values than the Brazilian older adults. In the simple and multiple logistic regression analyses, it was found that among Brazilian older adults, subjects with higher scores in the MMSE (OR 1.16; 95% CI 1.08–1.24), regardless of ADL performance (OR 2.13; 95% CI 1.31–3.47) and with scores 24–29 (OR 1.92; 95% CI 1.07–3.43) or 11–23 (OR 2.09; 95% CI 1.15–3.79) in neuroticism were more likely to assess their health as very good/good. On the other hand, the Portuguese older adults with intermediate 24–9 (OR 2.38; 95% CI 1.31–4.33) or low 11–23 (OR 5.31; 95% CI 2.69–10.45) scores in neuroticism were more likely to evaluate their health as very good/good.

**Conclusion:** Based on the findings of the present study and on the existing literature, it may be said that it is possible for people to age while keeping a positive perception of their own health, even in advanced old age; comparisons between the above-mentioned countries, however, point to the need for investments in healthcare systems so that older adults may enjoy greater physical independence and improved mental health.

## Introduction

Self-rated health represents a summary statement about the ways in which various aspects of health, both subjective and objective, are combined according to the subject's own perception (1). This variable is widely used in large population surveys on health and well-being, consolidating itself as a multidimensional indicator of health and a good predictor of adverse events in older adults (2). Answers to this assessment are influenced by social, cultural and personality aspects (3). Predictors of self-rated health include physical, socioeconomic, social, cognitive and emotional factors (4, 5).

Since the 1950s, studies have shown that, in old age, this assessment is associated both with indicators of well-being and with indicators of morbidity, functional decline and mortality (3). It greatly depends on how older individuals conceive of the notion of good physical health during aging, as well as on how they deal with feelings of control, autonomy and functionality in their daily lives (6).

The related literature presents well-documented relationships between self-rated health and gender, age, family configurations, and physical and psychological health status. Women live longer than men (7), but compared to them, they have disadvantages in several health indicators, including lower handgrip strength and higher prevalence of frailty. They also have greater dependence in activities of daily living and worse self-rated health than men do (8–11). Men who live alone are less likely to assess their self-rated health as poor than women who live with their spouses (12, 13). Harris et al. (14) observed a negative correlation between self-rated health and cognitive ability, neuroticism, frailty, longevity, depression and chronic diseases.

Personality traits are identified as important predictors of self-reported health. Research that used the inventory of five major personality factors (*The Big Five*) described by Costa and McCrae (15) revealed the existence of relationships between health predictors and all factors (Neuroticism, Extraversion-Intraversion, Openness to Experience, Agreeableness and Conscientiousness). However, among these, neuroticism is the factor that has been most closely associated with health status, including self-reported health, with clinical biomarkers, and with longevity (16). Higher scores in neuroticism are not only linked to negative subjective health assessments, but also to behavioral markers of loss of walking speed (17, 18) and of biological dysfunction (19). Notably, it is a widespread predictor of chronic respiratory disease, depression and dementia (20–22). Individuals with higher neuroticism scores may be more likely to negatively assess their own health than is suggested by objective indicators (19). The levels of perceived health remain stable with age, even in advanced old age, and are often higher than levels of health assessed through objective criteria (23). In addition, longitudinal studies have shown that there may be downgrades in self-rated health associated with objective normative or unexpected losses in a person's health status throughout the aging process (1, 23, 24), but that the scores of self-rated health tend to return to baseline levels as soon as recovery from previous health conditions occurs (10). Systematic decline in self-rated health with advancing age is associated with physical limitations, with disabilities, and with chronic pain and depression that often accompany the onset of disease, and not with the passage of time itself (1).

Self-rated health has been examined in different social and cultural contexts. In Brazil, we can highlight some relevant cross-sectional and longitudinal, population-based studies that evaluated self-assessed health: Projeto Bambuí (BHAS) (25), Health, Well-Being and Aging Study (SABE) (26), Frailty in Brazilian Older Adults (FIBRA) (27), and the Brazilian Longitudinal Study of Aging (ELSI-Brazil) (28). Results from a ten-year follow-up of the Bambuí older adult cohort (BHAS) showed a multidimensional structure regarding self-assessed health among older individuals, including socioeconomic status, social support, health status with emphasis on mental health, and access to healthcare services (29, 30). In the 2010 cohort of the SABE study, which started in 2000, prevalence of negative self-assessments was directly associated with worse indicators of income, education and consumer class (31). The FIBRA Study (2008–2009) identified multiple interactions of negative self-assessed health with indicators of structural, social and health vulnerability (27, 32), as well as of positive self-assessed health with maintenance of social engagement and of functional capacity (33, 34). ELSI-BRASIL baseline data, collected in 2015–2016, pointed out that worse self-rated health is directly associated with frailty (35, 36).

Relevant data were presented by the Study of the Aging Profile of the Portuguese Population (EPEPP), by the Epidemiology of Chronic Diseases (EpiDoc), and by Health, Aging and Retirement in Europe (SHARE). The EPEPP cross-sectional study (2005–2006), found that having autonomy, being independent in activities of daily living and being in good emotional state were related with positive self-assessment of health among long-lived individuals (37). EpiDoc data from the second wave of research collected between 2013 and 2015 revealed that the prevalence of multimorbidities combined with unhealthy lifestyles is associated with negative self-rated health among Portuguese older adults (38, 39). The Portuguese subsample of the SHARE study had negative self-assessed health, high prevalence of chronic diseases since early old age, high rates of depressive problems reported by women, and high levels of disability among the long-lived individuals (40). Scheel-Hincke et al. (10) examined gender differences in the performance of activities of daily living among older people from different European regions in a grouped sample of Waves 1 (2004–2005) through 6 (2015) of the SHARE Study. Differences in gender and in activities of daily living were found in all age groups for Southern Europe. Women had greater functional limitations and worse health outcomes. Serrano-Alarcón and Perelman (41) carried out a study with data from the SHARE study (Wave 4) for the countries of Southern Europe (Portugal, Spain and Italy). Results showed that among the subjects who composed the sample from these three countries the risk of suffering from severe functional limitation was higher among women and among individuals with low levels of schooling. The Portuguese sample showed relatively worse functional status and self-rated health when compared to the Spanish and Italian samples.

Self-rated health is, therefore, the most widely used indicator to assess individuals' overall health as well as to examine differences among groups within the same population and among different populations (42). The importance of cross-cultural studies stems from the fact that there are similarities and differences regarding human behavior, which cannot be explained without taking into account the complex context of cultural influences (43). Transcultural studies focus on biological, sociodemographic, environmental, health, social, cultural and psychological similarities and differences among diverse cultural and ethno-cultural groups.

The comparison between Portugal and Brazil may provide a unique perspective to shed light on the similarities and differences between the experience of subjective health and aspects of objective health in old age in both countries. Brazil and Portugal have markedly different dimensions—about 212 million vs. 10 million inhabitants, respectively—and are located in different continents—the former in South America and the latter in Europe. Despite this contrast, they share many cultural traits, including language, as a result of the Portuguese colonization of Brazil between 1500 and 1822. In 2020, the population aged 65 and over in Portugal represented 27% of the total population (44). Life expectancy at birth in Portugal is 81.12 years (44), 6 years higher than in Brazil, where it is estimated at 75.46 years (45). Brazilian older adults aged 65 and over represent 10.53% of the total population (46). Both countries face great challenges in terms of aging and health. In both, an increasing number of older people suffer from multiple chronic conditions, such as cardiovascular and cerebrovascular diseases, systemic arterial hypertension, rheumatic diseases, obesity and diabetes (26, 39, 47–51).

The perception of older adults about their health largely depends on the social and cultural context in which they live. The literature does not include comparative data about objective and subjective aspects of health, functional ability and personality in samples from Portuguese-speaking countries, such as Portugal and Brazil, that could contribute to understanding the extent and origins of these similarities and differences. The fact that Portugal is an aging country with a larger share of older adults in their population—much larger than Brazil's—may offer information that could support health policy planning in Brazil. Portugal is a country with sound investments in primary care. Their very low rates of hospitalization that could be avoided indicate the overall effectiveness of primary care services. In recent years, combined efforts to increase the efficiency of care have improved the cost-benefit ratio in the healthcare system's activity levels. On the other hand, low-income individuals in Portugal face a greater challenge in paying for medication and in having access to healthcare services not covered by the National Health System (SNS) (52). Differences in health literacy can also have an impact, although this is overlaid by issues related to internet access and health-related information available online, which can be difficult to access among the older population, as well as among people with lower education levels (52).

In Brazil, the mission of the Unified Health System (SUS) is to offer free healthcare services to all citizens, based on principles of equity and solidarity. Studies show that SUS has become more equitable between 1998 and 2008 (53) and that investments in primary care successfully reduced some aspects of social inequalities in Brazil between 2000 and 2012 (54). A major support service for SUS is the Elderly Caregiver Program (PAI), which covers only the city of São Paulo. PAI is presented as a challenge toward remodeling healthcare practices, as it particularly values care and focuses on providing services to older adults in situations of frailty. PAI is a modality of biopsychosocial home care for older individuals in situation of clinical frailty and social vulnerability, which provides services rendered by healthcare professionals and caregivers, for support and assistance in Activities of Daily Living (ADLs) and to meet other health-related and social needs (55, 56).

It must be noted that both countries have healthcare and home care policies for their older citizens; however, data from the present study may provide important information about self-rated health among older adults. The objectives of the present study are to explore common and distinctive characteristics of Brazilian and Portuguese older individuals aged 70 and over regarding self-rated health, according to sociodemographic variables, functional ability, independent performance of instrumental and basic activities of daily living, and neuroticism, as well as to analyse associations between positive self-rated health and these variables.

## Method

The present study was carried out based on the analysis of secondary data selected from electronic databases of two population studies involving samples from Portugal (*n* = 1,322) and Brazil (*n* = 1,284). The Portuguese sample from the DIA Study—From Disability to Activity: The Challenge of Ageing' (DIA; 2011–2014) -, with 1,322 individuals aged 55 and over, consisted of a probabilistic sample of men and women living in urban and rural areas of continental and island regions of Portugal, with total population of 10,374,822 at the time of the survey (2011–2014) (57). This sample of older adults was recruited in their places of residence and in areas where there is flow of people from this intended population, in randomly selected regions. To compose the sample of the Brazilian study—Frailty in Brazilian Older Adults (FIBRA)−418 older adults aged 70 and over who participated in the first follow up of the FIBRA study (2016–2017), whose baseline was carried out in 2008–2009, were selected. These older adults lived in the urban area of the city of Campinas (with 1,150,753 inhabitants in 2016), or in the urban area of the sub-district of Ermelino Matarazzo (117,372 inhabitants in 2016), one of the administrative regions of the city of São Paulo (20,579,717 inhabitants in 2016) (58). Subjects were recruited at home, based on a list of participants in the baseline study carried out in 2008 and 2009, with 1,284 older adults residing in 60 census tracts randomly drawn within the urban area of the two locations: 900 (60 census tracts) in Campinas and 384 (60 census tracts) in Ermelino Matarazzo.

Eligibility criteria for participants in the samples from the two studies were as follows: (a) 55 years and over in Portugal; 70 years and over Brazil; (b) permanent residence in the location, address and census tract; (c) in Portugal and in the FIBRA baseline study, sufficient sensory, physical function, health, cognition and language conditions to answer the instruments that would comprise the research protocol in each location. Subjects who were bedridden, terminally ill, undergoing cancer treatment, who had cerebrovascular accident sequelae, or who had advanced Parkinson's disease were excluded.

To compose the sample of the present comparative study, the following decisions were made in order to render samples as comparable as possible: (a) exclusion of rural-dwelling, island-dwelling and institutionalized subjects in the Portuguese sample, since the Brazilian sample was composed only of urban older adults living in family settings; (b) exclusion of older adults under 70 years of age from the Portuguese sample (*n* = 839), since the Brazilian sample had a minimum age for participation of 70 years; (c) in the Brazilian sample, exclusion of older adults who died between baseline and follow-up studies (*n* = 142), and of older subjects who were not located at the available addresses or who refused to participate in the study (*n* = 543); (d) selection of identical variables that included the same categories and the same answer criteria in the databases of the two countries; (e) selection of variables that had complete records of participant responses in the database of the two countries; (f) in both countries, exclusion of older adults who scored below the cut-off point in the Mini-Mental State Examination, based on current psychometric criteria in Portugal and in Brazil (scores of 23 or higher, out of 30, indicating normal cognition) (59–61).

**Figure 1:**
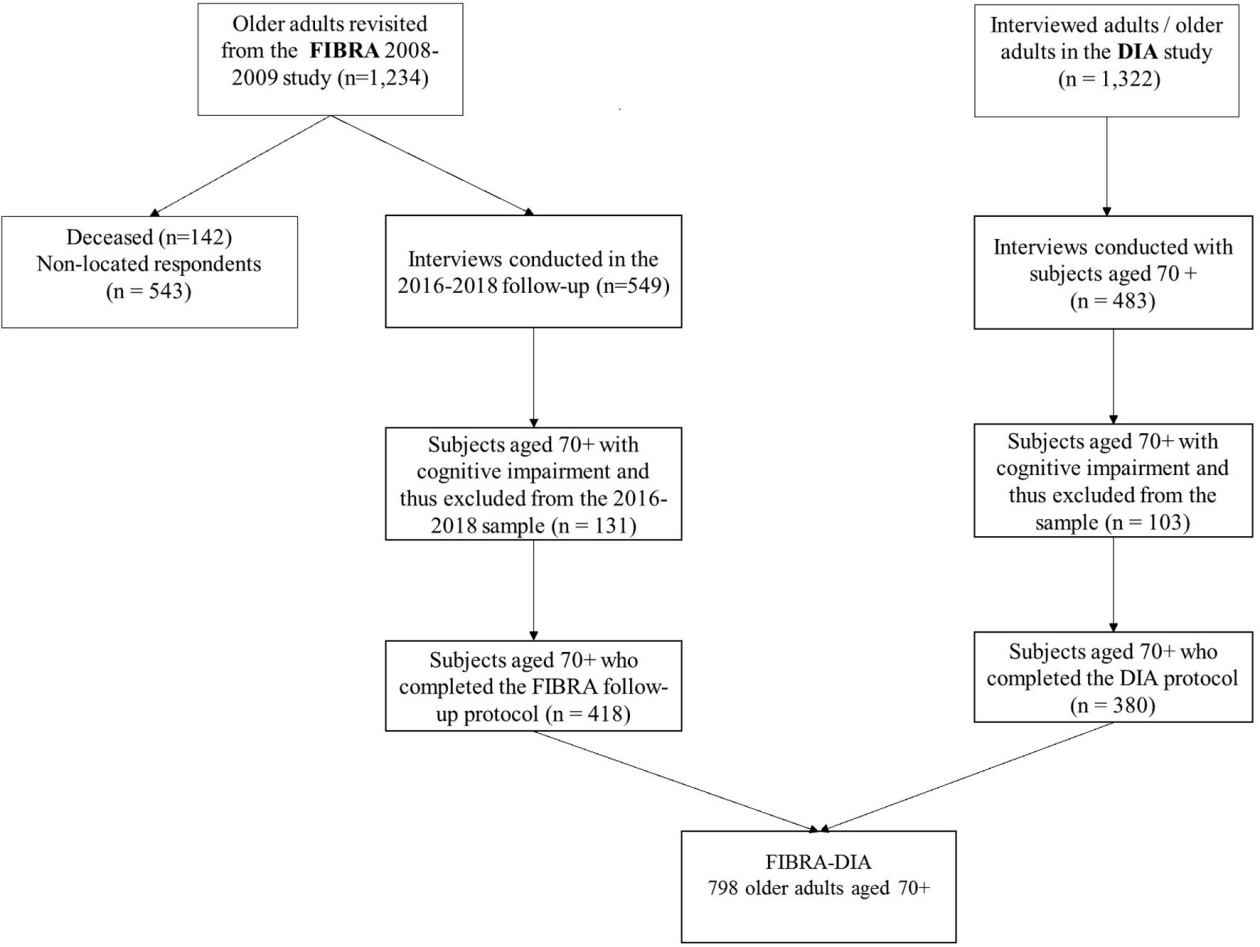
Sample flowchart.

As a result, the Portuguese sample consisted of 380 older adults aged 70 and over, and the Brazilian sample included 418 older adults aged 70 and over, living in urban areas either in Campinas city (*n* = 310) or in Ermelino Matarazzo (*n* = 108), a sub-district of the city of São Paulo (see Figure 1).

All procedures were conducted in accordance with ethical precepts regarding research with human subjects, as provided for in the Helsinki Convention, in the Brazilian National Health Council and in the principles of the European Code of Conduct for Research Integrity, thus avoiding fabricated data, falsification, plagiarism or any other research misconduct. Participants signed an informed consent form prior to the interview. The FIBRA Study was approved by the Ethics Committee of the State University of Campinas, Brazil, CAAE registry numbers 49987615.3.0000.5404 and 92684517.5.1001.5404. The DIA Study was approved by the Ethics Committee of the Unit for Research and Training on Adults and the Elderly | UNIFAI at the Abel Salazar Institute of Biomedical Sciences of University of Porto | ICBAS- UPORTO.

## Variables and Measures

Sociodemographic: In both studies, the following variables were examined: gender, with “male” and “female” as options; age (through a question about date of birth, which was compared with the date of the interview to obtain the chronological age); and whether the person lived alone (“yes” or “no”). In the FIBRA Study, education in terms of years of schooling was categorized into “never went to school,” “1 to 4 years,” “5 to 8 years” and “9 or more years of schooling.” In the DIA Study, the answers to the same question were categorized into “up to 8 years” and “9 or more years of schooling.”

### Cognitive Status

In both studies, individuals' total scores in the Mini-Mental State Examination (59–61) were considered as a continuous variable.

### Handgrip Strength

Widely used as an indicator of objective functional ability, this variable was measured using a Jamar hydraulic dynamometer (Jamar, Lafayette Instruments, Lafayette, Indiana, USA) or Support/GRIP placed in the participant's dominant hand in Brazil and in alternating hands in Portugal. In the Brazilian sample, three attempts were made with a 1-minute interval between them. In the Portuguese sample, there were four attempts interspersed with 30-s intervals. In the two studies, the average was calculated and measures below the first quintile of the distribution were considered as “low grip strength.” Both were adjusted for gender and body mass index [BMI = weight/height squared (kg/m2)].

### Independent Performance of BADL and IADL

In both countries this variable was investigated through reports from participants on the level of help needed for the performance of two classes of activities: Basic Activities of Daily Living (BADLs) and Instrumental Activities of Daily Living (IADLs). The first—Basic Activities of Daily Living—are related to basic survival and include eating, bathing, getting dressed, toileting, and functional mobility (62, 63) and the second—Instrumental Activities of Daily Living—are related to practical life, like using the phone, traveling, shopping, cooking, housekeeping, managing medication and managing money (63, 64). In both classes of activities, each item had as possible responses independent for carrying out the task' and “partially or totally dependent on assistance.” Scores > 1 in the IADL and BADL inventories were considered as suggestive of dependency for each of the classes.

### Neuroticism

The Five-Factor Model of Personality, or Big Five, is regarded as a psychometrically valid representation of an individual's personality trait structure and is certainly the most widely used instrument for personality assessment. The questionnaire in this model is the NEO-PI Five-Factor Inventory (65), comprising 300 items with a scale of five points each (from strongly agree to strongly disagree) to assess Neuroticism, Extraversion-Intraversion, Openness to Experience, Agreeableness and Conscientiousness. While the DIA Study applied an abridged version (60 items) of this inventory, semantically and culturally adapted to Portuguese (66), the FIBRA Study chose to apply the 12 items corresponding to Neuroticism (67), the most frequently studied factor in older adult populations and one that has well-established correlations with multimorbidity, mortality, disability, depression, and low levels of subjective well-being in this population (68).

In both studies, answers were categorized into ranges of respondent scores: numerical values 30–48, indicating high level of neuroticism; 24–29, indicating intermediate level of neuroticism; and 11–23, indicating low level of neuroticism.

### Self-Rated Health

Self-rated health was measured both in the FIBRA and DIA studies using a five-point Likert scale (1—very poor, 2—poor, 3—fair, 4—good and 5—very good) (69, 70). Answers were categorized into “very good/good” and “fair/poor/very poor.”

## Statistical Analysis

To describe the profile of the sample for the studied variables, frequency tables of the categorical variables (gender, age group, education) were drawn up, with absolute frequency values (*n*) and percentages (%), and with descriptive statistics of the numerical variables (age, handgrip strength, Mini-Mental State Examination), with mean values, standard deviation, minimum and maximum values, median and quartiles. The comparison of categorical variables between two groups was performed using the chi-squared or Fisher's exact tests. To compare numerical variables between two groups, the Mann–Whitney test was used due to the absence of a normal distribution of the variables. To study the factors associated with positive self-rated health, logistic regression analysis was performed. Variables that were associated with self-rated health were included in the models in the simple analysis with statistical significance indicated by *p* ≤ 0.20. In the final model, associations with statistical significance indicated by *p* < 0.05 were selected.

## Results

The sample consisted of 418 Brazilian and 380 Portuguese older adults, of which more than 60% were women. There were statistically significant differences related to the proportion of participants aged 70 to 79 in the two samples: in Portugal, individuals aged 70 to 79 prevailed ($\bar{\text{X}}$ = 76.80 ± 5.28), whereas in Brazil there was a higher proportion of octogenarians ($\bar{\text{X}}$ = 80.31 ± 4.67). In Portugal there was a higher percentage of older adults living alone than in Brazil, where more including family arrangements predominated. There were more individuals with no dependence for the performance of BADLs in Brazil than in Portugal, and more individuals with dependence on at least one BADLs in Portugal than in Brazil. However, in both samples the vast majority of the older adults were independent when performing ADLs. In Portugal, there was a higher percentage of older individuals who scored high for neuroticism than intermediate or low, whereas in the Brazilian sample there were more individuals at the intermediate level than at the low or high levels (Table 1). The Portuguese older adults had higher average total scores in the MMSE ($\bar{\text{X}}$ = 27.52% ± 2.1 against = 24.94 ± 2.9 of the Brazilians); in handgrip strength, older Portuguese had higher mean strength ($\bar{\text{X}}$ = 22.85 ± 7.3) than older Brazilians ($\bar{\text{X}}$ = 22.73 ± 11.1); in neuroticism, higher values were recorded among the Portuguese subjects ($\bar{\text{X}}$ = 32.05 ± 4.0) than in the Brazilian sample ($\bar{\text{X}}$ = 24.89 ± 7.1.0).

**Table 1:** Sample characteristics according to country (*n* = 798).

	**Total** ** (*n* = 798)**	**Portugal** ** (*n* = 380)**	**Brazil** ** (*n* = 418)**	***P*-value**
	***n* (%)**	***n* (%)**	***n* (%)**	
**Gender**				
Female	552 (69.2)	260 (68.4)	292 (69.9)	0.661
Male	246 (30.8)	120 (31.6)	126 (30.1)	
**Age (years)**				
70–79	457 (57.2)	274 (72.1)	183 (43.8)	** <0.001**
≥ 80	341 (42.8)	106 (27.9)	235 (56.2)	
$\bar{\text{X}}$ ± SD	78.59 ± 5.17	76.80 ± 5.28	80.31 ± 4.67	
Handgrip strength, ($\bar{\text{X}}$ ± SD)	22.79 ±9.6	22.85 ± 7.3	22.73 ± 11.1	0.030
**Living alone**				
Yes	212 (29.6)	130 (41.0)	79 (19.7)	**<0.001**
No	505 (70.4)	187 (59.0)	321 (80.2)	
**Education**				
No schooling	111 (14.0)	53 (14.2)	58 (13.9)	0.691
1 to 8 years	582 (73.6)	270 (72.4)	312 (74.6)	
≥ 9 years	98 (12.4)	50 (13.4)	48 (11.5)	
**Basic activities of daily living**				
Dependent in 1 or more	219 (28.5)	123 (34.8)	96 (23.1)	**<0.001**
Independent	550 (71.5)	230 (65.2)	320 (76.9)	
**Instrumental activities of daily living**				
Dependent in 1 or more	388 (49.3)	181 (49.1)	207 (49.5)	0.895
Independent	399 (50.7)	188 (50.9)	211 (50.5)	
**Neuroticism**				
30–48	230 (31.0)	158 (48.5)	72 (31.3)	**<0.001**
24–29	298 (40.1)	111 (34.0)	187 (44.8)	
11–23	215 (28.9)	57 (17.5)	158 (37.9)	
**Assessment of overall health**				
Very poor/poor/fair	474 (60.3)	276 (75.0)	198 (47.4)	**<0.001**
Good/very good	312 (39.7)	92 (25.0)	220 (52.6)	

*P <0.05 value with statistical significance; Pearson's Chi-Squared Test*.

*Values in bold are p values <0.05*.

For the Portuguese older adults, a higher prevalence of self-rated health as very good / good was observed among men with ≥ 9 years of schooling, with higher average scores in the MMSE and in handgrip strength, and who were independent in carrying out activities of daily living and had low neuroticism scores when compared to the reference categories. As for Brazil, it was observed that older adults with higher average scores in the MMSE, who were independent in carrying out activities of daily living and with low neuroticism scores had a higher prevalence of very good / good self-rated health (Table 2).

**Table 2:** Comparison of samples from Brazil and from Portugal regarding self-rated health as “good” or “very good,” considering sociodemographic variables, independent performance of basic and instrumental activities of daily living and neuroticism.

**Variables**	**Brazil**		**Portugal**	
	***n* (%)**	*P*-value^*^	***n* (%)**	*P*-value^*^
**Age**				
70–79 years	96 (52.5)	0.950	66 (24.5)	0.734
≥ 80	124 (52.8)		26 (26.3)	
**Gender**				
Female	148(50.7)	0.225	54 (21.5)	**<0.001**
Male	72 (57.1)		38 (32.5)	
**Education**				
No schooling	22 (37.9)		12 (23,5)	**<0.001**
1–8 years	171 (54.8)	0.053	56 (21.2)	
≥9 years	27 (56.3)		23 (50.0)	
Handgrip strength	22.06 ± 9.2	0.618	24.56 ± 8.4	0.023
**Living alone**				
Yes	43 (54.4)	0.738	31 (25.0)	0.978
No	168 (52.3)		46 (25.1)	
**Basic activities of daily living**				
Dependent in 1 or more	36 (37.5)	**0.001**	19 (15.4)	**0.002**
Independent	183 (57.2)		70 (30.6)	
**Instrumental activities of daily living**				
Dependent in 1 or more	95 (45.9)	**0.006**	29 (16.1)	**<0.001**
Independent	125 (59.2)		63 (33.7)	
**Neuroticism**				
30–48	27 (37.5)	**0.016**	24 (15.4)	**<0.001**
24–29	103 (55.1)		33 (30.3)	
11–23	90 (57.0)		28 (49.1)	

* *Pearson's chi-squared test; statistically significant differences if p <0.05*.

*Values in bold are p values <0.05*.

In the simple logistic regression analysis, the variables education, cognitive status, independence in the performance of IADLs and BADLs, and neuroticism were selected as significantly associated with positive self-rated health among Brazilian older adults. In the adjusted analysis, Brazilian older adults with the highest MMSE scores (OR 1.16; 95% CI 1.08–1.24), independent to perform ADLs (OR 2.13; 95% CI 1.31–3.47) and with scores 24–29 (OR 1.92; 95% CI 1.07–3.43) or 11–23 (OR 2.09; 95% CI 1.15–3.79) in neuroticism were more likely to assess their health as very good / good than those who had lower scores in the MMSE, those who needed more assistance to perform ADLs and those who had higher scores in neuroticism (see Table 3).

**Table 3:** Results of simple and multiple logistic regression analyses for self-rated health as “good” or “very good” in the Brazilian sample.

**Variable**	OR (95% CI)^*^ simple	**OR (95% CI)** adjusted^**^
Age ≥80 years (ref: 70–79 years)	1.01 (0.68–1.49)	
Male (ref: female)	0.77 (0.50–1.17)	
Education in years of schooling (ref: no schooling)		
1–8 years	1.98 (1.11–3.52)	
≥9 years	2.10 (0.96–4.58)	
Living alone (ref: yes)	0.99 (0.98–1.00)	
Handgrip strength (continuous variable)	1.47 (0.97–1.02)	
MMSE (continuous variable)	1.15 (1.08–1.23)	1.16 (1.08–1.24)
IADLs with dependence (ref: independence)	1.71 (1.16–2.52)	
ADLs with dependence (ref: independence)	2.22 (1.39–3.55)	2.13 (1.31–3.47)
**Neuroticism** (ref: 30–48)		
24–29	2.04 (1.17–3.56)	1.92 (1.07–3.43)
11–23	2.20 (1.24–3.90)	2.09 (1.15–3.79)

* OR (Odds Ratio) = odds ratio for self-rated health as “good” or “very good”; 95% CI OR = 95% confidence interval for the odds ratio; Ref: Reference;

** *OR adjusted by the variable's education, MMSE, ADLs, IADLs and Neuroticism*.

Gender, education, cognitive status, handgrip strength, independent performance of ADLs and neuroticism were the variables selected to enter the multiple regression model for Portuguese older adults. In the adjusted model, subjects with intermediate scores 24–29 (OR 2.38; 95% CI 1.31–4.33) or low scores 11–23 (OR 5.31; 95% CI 2.69–10.45) in neuroticism were more likely to evaluate their health as very good/good than those that scored in the highest range (Table 4).

**Table 4:** Results of simple and multiple logistic regression analyses for self-rated health as “good” or “very good” in the Portuguese sample.

**Variable**	OR (95% CI)^*^ **simple**	**OR (95% CI)** adjusted^**^
Age ≥80 years (ref: 70–79 years)	1.09 (0.64–1.85)	
Male (ref: female)	0.56 (0.34–0.93)	
Education in years of schooling (ref: no schooling) ≥9 years 1–8 years	3.25 (1.36–7.73) 0.87 (0.42–1.78)	
Living alone (ref: yes)	0.99 (0.99–1.00)	
Handgrip strength (continuous variable)	1.00 (0.99–1.02)	
MMSE (continuous variable)	0.99 (0.97–1.01)	
IADLs with dependence (ref: independence)	2.64 (1.60–4.36)	
ADLs with dependence (ref: independence)	2.40 (1.37–4.23)	
Neuroticism [ref: 30–48] 24–29 11–23	2.38 (1.31–4.33) 5.31(2.69–10.45)	2.38 (1.31–4.33) 5.31 (2.69–10.45)

* OR (Odds Ratio) = odds ratio for self-rated health as “good” or “very good”; 95% CI OR = 95% confidence interval for the odds ratio; Ref: Reference;

** *OR adjusted by the variables education, MMSE, ADLs, IADLs and Neuroticism*.

## Discussion

The present study aimed to examine common and distinctive characteristics of Brazilian and Portuguese older individuals aged 70 and over regarding self-rated health, according to sociodemographic variables, functional ability, independent performance of instrumental and basic activities of daily living, and neuroticism, as well as to analyse associations between positive self-rated health and these variables. According to the multiple regression analysis, having a high score in MMSE, being independent for performing ADLs, and having intermediate or low levels of neuroticism are associated with self-rated health as “very good” or “good” among Brazilian older adults. Low level of neuroticism was found to be a major determinant for Portuguese older adults to assess their health as “very good” or “good.”

There was prevalence of women and of participants with 1 to 8 years of schooling in samples from both countries, replicating data from their respective populations. Brazilian women aged 70 and over represent 3.7% against 2.7% of men in the total Brazilian population (71). In Portugal, the representation of women aged 70 and older is also higher (9.61%) compared to that of men (6.50%) (44). Feminization of old age is a recognized phenomenon worldwide and is due to the decrease in mortality rates of women in relation to men's (72). Women are the majority in the older adult population in all regions of the world. It is estimated that they live, on average, 5 to 7 years longer than men (73). However, along their lives, women accumulate disadvantages such as violence, discrimination, lower wages than men, double shifts, low level of education, loneliness due to widowhood, poverty and dependence on external resources (74). In old age, one form of assistance for women is their support networks, which in many cases are composed of family members who fulfill the needs that government programs do not (72). In return, they also provide support to their family members, as they are often providers through their retirement payments, pensions and other benefits. These women play an important social role as grandparents and caregivers, providing help to sick friends or neighbors and taking on more and more responsibilities (75).

Difficulties in having access to education was evidenced in other Brazilian studies (36, 76, 77). In Portugal, the level of schooling of older adults is much lower than the level of schooling of the adult population across the country. Average years of schooling is 6.3, which means that most older adults did not have access to high school or have stopped studying before that. In addition, women have even lower levels of schooling (40, 41, 78). As a rule, education is built early in life and affects a person's future health in many aspects. People with more years of schooling tend to have higher incomes, safer occupations, more self-esteem, more adherence to healthy behaviors and more resources for timely healthcare seeking (79, 80).

Sociodemographic differences are related to age groups and to living alone. Although Portugal is characterized as a very aged country with a high percentage of older adults, and although about 5.9% of its population is aged 80 and over (40, 78), in the present study people between 70–79 years of age were more prevalent, similarly to what was observed in investigations conducted by other recent studies involving Portuguese older individuals (39, 40, 78). The Portuguese older adults are more prone to being institutionalized than their Brazilian counterparts (81). About 3.63% of the population of older adults live in Residential Facilities for Older Adults (ERPI) in Portugal, whereas a little over 1.0% of the Brazilian older adults are institutionalized, in part due to the absence of operative policies and resources concerning oldest older adults, in part due to prejudice against institutionalization in Brazil. About 60% of this population live alone or with other older adult(s) (44). In Brazil, 15.3% of the older adult population live alone (82). Older adults not living alone (84.9%) often live in family arrangements typical of poverty, which include one or two older individuals, children, grandchildren and other relatives, and they tend to be important providers of financial support for their families (69.8% of their family income) (83).

Handgrip strength is an indicator of intrinsic capacity, which affects physical and cognitive independence. It is used to identify phenotypes of sarcopenia and frailty, and it is a potential predictor of mortality in older adults (84). Mental state is used to identify and monitor cognitive alterations (85). Studies conducted by Mendes et al. (86) and by Vaz-Patto et al. (87) indicated that cognitive scores have a positive correlation with handgrip strength. Decreased handgrip strength is strongly associated with development of mild cognitive impairment, especially in people aged 80 and over (86). The Brazilian sample includes subjects aged 80 and over, which suggests a higher risk of adverse health events. Results similar to those of the present study were observed by studies (76, 88–90) regarding Brazilian octogenarians.

Portuguese older adults showed a higher level of dependence in ADLs. Previous studies with data from the Survey of Health, Aging and Retirement in Europe (SHARE) found similar results. More than 20% of people in Southern Europe (Spain, Portugal and Italy) over 50 years of age suffer from limitations and functional dependencies. Country, gender and socioeconomic status were pointed out as important predictors of severe functional dependence. Functional limitations were higher among Portuguese older adults, especially women with low level of schooling (41). Women live longer than men, but compared to them, they have disadvantages in several health indicators, including lower handgrip strength and higher prevalence of frailty. They have greater dependence in activities of daily living and worse self-rated health than men do (9, 10, 91). European women have a higher risk of having limitations in ADLs than European men, and these gender differences increase with age. Dependence in performing ADLs was found in all age groups in Southern Europe, and in the 65–79 and in the 80 and over age groups in Western and Eastern Europe (10).

Higher values in neuroticism and in “very poor,” “poor” or “fair” self-rated health were identified among the Portuguese older adults. Neuroticism involves individual differences in the tendency to experience negative emotions and is correlated with self-reported health problems, but not with objective measures of health (92). Older adults with higher levels of neuroticism reported more health problems, are more prone to display negative affects, have negative self-rated health and high levels of hypochondria, as found in the research conducted by Morgan et al. (93). Low scores in conscientiousness and increased neuroticism indicated worse self-rated health in two assessments 10 years apart in the Midlife in the United States Survey (MIDUS) (94). Reduced conscientiousness and extroversion and increased neuroticism predicted worse self-reported physical health in the study The Household, Income and Labor Dynamics in Australia (HILDA) (95). The authors also found that associations between changes in personality traits and health and neuroticism were stronger in cohorts aged 70 and over.

Although the Brazilian sample is older and has lower handgrip strength scores, it is more independent in ADLs and has lower levels of neuroticism than the Portuguese sample; twice as many Brazilian participants have a positive self-assessment of health (52.6 vs. 25% of Portuguese older adults). This difference in prevalence may be related to contextual variables, such as the quality of existing social support networks (37.77) and individual psychological factors such as neuroticism, suggesting that the Portuguese older adults might have a more critical and more demanding attitude. Self-rated health among Portuguese older adults was explored in a recent study conducted by Alarcão et al. (96). Results showed that, on average, older community-dwelling Portuguese evaluate their overall health status as “fair.” The 2014 European Social Survey, which contains data from 19 countries, reports that the best overall self-rated health was that of Ireland, and the worst was that of Portugal (97).

Positive self-rated health is an excellent indicator of one's own health, predicting the survival of each individual (98). It is related to good physical, cognitive and emotional components, in addition to a feeling of well-being and satisfaction with life (3, 99). Self-rated health is related to some important aspects of the health of older adults, such as functional capacity, physical condition and mental health (100); and to sociodemographic and economic characteristics, such as gender, schooling, housing arrangements and income (8).

The dissociation between objective health status and self-rated health is discussed in the related literature as one of the manifestations of the so-called paradox of successful aging, even in the presence of objective losses and limitations (8.23). Although there is an increase in chronic diseases, a decrease in physical and cognitive abilities, and changes in psychosocial and cognitive-emotional state in old age, the effects of functional reserves and of gains in other aspects of life have a compensatory effect on the functioning of older individuals. One example of this effect is their positive perception of health, even in the presence of losses and limitations (101, 102). Another example is that an increase in the level of disability does not necessarily lead to lack of confidence and determination, because people are characterized by a sense of well-being and pride in their status as survivors (50). Furthermore, older adults compare themselves to other people who are worse off, highlighting the compensatory effects of perceptions of the self (103).

The literature suggests that gender differences vary among different health measurements and assessments, age cohorts, time periods and geographic regions (104). Data from the Survey of Health, Aging and Retirement in Europe (SHARE) indicate that in all regions of Europe men assess their own health more positively than women and have lower risks of dependence in activities of daily living than women do (10). High levels of schooling among men suggest better self-rated health and higher levels of functional capacity and cognitive performance in the Southern Europe sample from SHARE. Having more years of schooling means having access to health information, thus favoring healthier behaviors and better lifelong work opportunities, with access to healthcare services (41, 96).

Cognitive scores have a positive correlation with handgrip strength (87). In general, the values for handgrip strength of older men are higher than those of women (8). In a study conducted by Mendes et al. (86) with a sample of 1,500 Portuguese older adults, age, cognitive status and physical activity were significantly correlated with handgrip strength for both genders, with higher values among men.

Among the Brazilian older adults, self-rated health as “very good” or “good” is related with higher mean values in the MMSE, low levels of neuroticism and independence in ADLs. Based on the longitudinal study Health, Well-being and Aging | SABE (31), more than 48% of the older adults in a Brazilian sample assessed their health as “good” or “very good” in the 10 consecutive years of that survey. Positive self-perceived health was also associated with high cognitive status, functional independence and less depressive symptoms in a study conducted by Krug et al. (90) in a regional sample of older Brazilians. Maintained functional capacity was the variable with the greatest explanatory potential for self-rated health, according to microdata from the National Health Survey (2013), conducted by Figueiredo et al. (105) and by Zanesco et al. (106) with a sample (23.815) of older individuals (60 to 109 years of age) from all regions of Brazil. The importance of physical functionality and its influence on self-assessment of health was also confirmed by a study by Belmonte et al. (34) with a sample of community-dwelling older adults.

In a comparative study conducted by Xu et al. (2) with data from HRS and CHARLS, the authors found that 78% of older adults in China reported fair or poor health status, whereas almost 74% of older adults in the USA reported it as excellent, very good or good. However, older individuals who reported more limitations in activities of daily living, worse self-reported memory, worse mental health, and chronic health conditions had worse self-rated health in both countries.

Adjusted analysis of the simple and multiple logistic regression model indicated that higher scores in the MMSE, being independent in the performance of ADLs and low scores in neuroticism had a strong correlation with “very good” or “good” self-rated health among Brazilian older adults. Low level of neuroticism was found to be a major determinant for Portuguese older adults to assess their health as “very good” or “good.” In Portugal, only neuroticism was associated with “very good” or “good” self-rated health among the older subjects. According to Jylhä's (4) self-rated health paradigm (2009), personal psychological factors, such as affective dispositions, create an important framework for evaluating health components and, as a result, significantly influence people's assessments of their own health. The tendency to experience positive or negative affects is strongly related with personality traits, especially to extroversion and to neuroticism (107, 108); personality traits can predict results regarding good or bad self-assessed health (109). Low levels of neuroticism in old age may suggest good outcomes in physical health and in cognitive functioning (110).

The present study has strengths and limitations. Among its strengths is the fact that this study presents pioneering data on positive self-rated health by older adults aged 70 and over from two Portuguese-speaking countries, and is useful as an examination of the possibility of conducting comparative research with controlled samples from Brazil and Portugal. Most of the studies available on self-rated health are more focused on groups of young people or of young older adults. Little research is conducted on looking into the variables associated with self-rated health, especially positive, among oldest older adults. As individuals age, their standards of what they consider to be good health are adjusted in the presence of physical and emotional decline; consequently, findings from research on younger people do not apply to older adults, thus highlighting the importance of carrying out specific studies with older participants. Among the limitations of this study are its relatively modest sample size and the fact that it was based on samples and measurements that were not specifically designed for it. Another aspect is related to the fact that older people aged 80 and over have a higher share in the Brazilian sample, even though the aging pattern of the Portuguese population includes more oldest older adults. The sample base of the FIBRA study is representative of cohorts 70–79 years old and 80 and over, whereas the base of the DIA study includes people aged 55 and over.

It is possible to have a positive perception of one's health even in advanced old age. The present paper shows that older people from two different countries, and particularly Brazilians, often rated their own health as “very good” or “good.” There are important differences between Brazil and Portugal, both in the prevalence of positive perceptions of one's own health and in its impact on mental and physical health. Results from comparisons between countries suggest a need for investing in improving one's health throughout life, because childhood living conditions can be considered distal determinants of independent performance of activities of daily living and of reduced burden of chronic diseases among older adults (111, 112). Personality, expressed through the trait neuroticism, deserves special attention. Neuroticism is a predictor of many different mental and physical disorders, including comorbid conditions, and of the frequency of use of mental health and general healthcare services (113). Nevertheless, “healthy neuroticism” can be a good thing: if neurotic people channel their concerns into proactive healthcare behaviors, such as promptly seeking medical care, it can substantially improve treatment prognosis (114). It should be emphasized that healthcare systems need to be designed to meet these demands as people age. In Portugal, population aging is considered one of the main sources of pressure on the National Healthcare Service (SNS). Patients aged 70 and over represent 50.53% of hospitalization costs (115). In Brazil, 75.3% of older adults rely exclusively on the services provided by the Unified Health System (SUS), and this represents 30% of hospitalization costs (36).

The data provided by the present study point to the importance of public health policies and to the need for integrated healthcare practices with regards to aging populations. Portugal has considerable experience in caring for its very long-lived population, with increasing healthcare needs and chronic conditions, since it is experiencing a continuous and growing population aging, long before that same trend might be experienced in Brazil. It has strong primary care systems and preventive services that result in lower mortality rates than the European Union average (with 140 per 100,000 inhabitants in 2016, compared to 149 in 2011) (52). The social responses with greater representativeness in the scope of services and equipment networks that aim to support older adults are represented by the Residential Structures, the Day and Night Centers and the Home Support Service (116).

These results highlight health inequalities among older adults in both countries, which require enhanced care systems and investments in policies to promote physical and mental health. Older adults prone to psychological suffering, with unrealistic ideas, excessive impulses and poor adaptive coping responses, hypochondriacs, characteristics of neuroticism, and older adults with functional dependencies need special attention and specialized services, which seem to be insufficient both in Brazil and in Portugal.

It should be noted that healthcare policies must be devised to play an important role in reducing social inequalities in healthcare in Brazil and in Portugal, with planning that guarantees care for vulnerable populations, including that of older adults. This can be accomplished: (1) through greater investments in the area of primary care; (2) through the reorganization of mental healthcare services for easier access by older adults; (3) by expanding the dissemination of good healthcare practices, with home visits by healthcare professionals particularly in the most vulnerable communities; and (4) by ensuring that everyone has access to free services, based on principles of equity and solidarity.

Finally, a set of preventive measures is especially relevant for older populations, as they tend to have more healthcare needs, lower incomes due to retirement or to the inability to continue working, and may have more difficulties compared to younger cohorts for managing complex healthcare issues involving technological resources.

## Data Availability Statement

The original contributions presented in the study are included in the article/supplementary material, further inquiries can be directed to the corresponding author/s.

## Ethics Statement

The studies involving human participants were reviewed and approved by Scientific Research Ethics Committee of the Faculty of Medical Sciences of the State University of Campinas and Ethics Committee of the Unit for Research and Training on Adults and the Elderly | UNIFAI of the Abel Salazar Institute of Biomedical Sciences at the University of Porto | ICBAS-UPORTO. The patients/participants provided their written informed consent to participate in this study.

## Author Contributions

MC, AN, and CP contributed to the conception and design of the present study. GC organized the database, organized the tables and formatted the manuscript. FB performed the statistical analyses and organized the tables. MC wrote the manuscript. All authors contributed to the review of the manuscript, reading and approving the submitted version.

## Conflict of Interest

The authors declare that the research was conducted in the absence of any commercial or financial relationships that could be construed as a potential conflict of interest.
